# The Rate of Cisplatin Dosing Affects the Resistance and Metastatic Potential of Triple Negative Breast Cancer Cells, Independent of Hypoxia

**DOI:** 10.3390/pharmaceutics14102184

**Published:** 2022-10-13

**Authors:** Omkar Bhatavdekar, Inês Godet, Daniele Gilkes, Stavroula Sofou

**Affiliations:** 1 Department of Chemical and Biomolecular Engineering, The Johns Hopkins University, Baltimore, MD 21218, USA; 2 Johns Hopkins Institute for NanoBioTechnology, The Johns Hopkins University, Baltimore, MD 21218, USA; 3 Department of Oncology, The Sidney Kimmel Comprehensive Cancer Center, The Johns Hopkins University School of Medicine, Baltimore, MD 21231, USA; 4 Cellular and Molecular Medicine Program, The Johns Hopkins University School of Medicine, Baltimore, MD 21231, USA; 5 Cancer Invasion and Metastasis Program, The Johns Hopkins University School of Medicine, Baltimore, MD 21231, USA

**Keywords:** triple negative breast cancer, cisplatin, hypoxia, nanoparticle, diffusion, solid tumors, rate of drug release, metastatic potential

## Abstract

To best control tumor growth and/or metastasis in triple negative breast cancer (TNBC), it may be useful to understand the effect(s) of chemotherapy delivery (i.e., the rate and pattern of exposure to the drug) on cell sub-populations that have experienced different levels of hypoxia (and/or acidosis). In this spirit, MDA-MB-231 TNBC cells, and their hypoxia-reporter counterparts, were characterized for their sensitivity to cisplatin. When in the form of multicellular spheroids, that capture the diffusion-limited transport that generates hypoxic and acidic subregions within the avascular areas of solid tumors, the effects of the rate and pattern of exposure to cisplatin on cell viability and motility/migration potential were evaluated for each cell sub-population. We demonstrated that cell sensitivity to cisplatin was not dependent on acidosis, but cell resistance increased with exposure to hypoxia. In spheroids, the increase of the rates of cell exposure to cisplatin, at a constant cumulative dose, increased sensitivity to chemotherapy and lowered the cells’ metastatic potential, even for cells that had experienced hypoxia. This effect was also shown to be caused by nanocarriers engineered to quickly release cisplatin which deeply penetrated the spheroid interstitium, resulting in the fast and uniform exposure of the TNBC tumors to the agent. This rate and dosing-controlled model may effectively limit growth and/or metastasis, independent of hypoxia. This mode of chemotherapy delivery can be enabled by engineered nanocarriers.

## 1. Introduction

Treatment of advanced triple negative breast cancer (TNBC) remains one of the unmet needs in global healthcare over the last few decades [1,2]. Clinical outcomes for advanced TNBC still remain devastatingly poor with median overall survival ranging from 8 to 13.3 months [3]. Despite the latest advances in immunotherapy [4] and radiotherapy [5], following surgery, chemotherapy [6,7] remains the frontline option for treating both the primary tumors and recurrence in TNBC due to a lack of reliable cell surface markers for molecularly targeted therapies [8]. Therapeutic approaches to treat TNBC have failed due to their inability to reduce the metastatic potential of the disease and/or to effectively handle the induced resistance to chemotherapy.

Cisplatin (CDDP) has been used as a frontline chemotherapeutic for multiple solid tumors including TNBC [9,10,11,12]. CDDP’s mechanism of action primarily entails the formation of platinum-DNA adducts by binding to the genomic and/or mitochondrial DNA [12] inhibiting DNA replication, which finally leads to apoptosis or necrosis. Although CDDP is extensively used in the clinic, CDDP has major safety considerations, with nephrotoxicity and hepatotoxicity being the primary adverse side-effects [13,14]. These side effects limit the maximum tolerated dose (MTD), which may result in incomplete tumor killing, causing major setbacks on the clinical outcomes. Although there are now second and third generation platinum-based agents, such as carboplatin and oxaliplatin used in the clinic globally, the latter have not been able to markedly improve the therapeutic index [15].

One of the primary reasons for TNBC’s lethality is chemoresistance contributing to a poorer prognosis; this has been associated with the prevalence of hypoxia within the established, soft-tissue solid (primary and/or metastatic) tumors. To make the prognosis even worse, the evidence shows that cells that experience hypoxia in the primary tumor have a higher metastatic potential and migrate to distant organs, such as the lungs [16] and/or the brain [17], causing cancer’s deadlier recurrence. Although there are a variety of debated mechanisms for how hypoxia affects the tumor landscape, it is well established that hypoxia inducible factor 1 and 2 (HIF-1/2), hypoxia-regulated transcription factors, modulate the expression of hypoxia-inducible genes including, but not limited to, the growth factor regulating genes, tumor survival genes, etc. [18].

Along with metabolic imbalances, hypoxia is accompanied by acidosis [19] which results in a lowered pH in the tumor microenvironment, due to anaerobic glycolysis and a slower clearance of acidic metabolites. This is not just detrimental for the local tumor microenvironment; it has also been hypothesized that acidosis accelerates the late steps in carcinogenesis resulting in the degradation of the extracellular matrix (ECM), potentially increasing the intravasation of tumor cells into the bloodstream and, therefore, contributing to metastasis [20].

The above factors occurring in the solid tumor microenvironment, may promote the development of multiple cancer cell subpopulations, each with a different evolution landscape, resulting in different drug sensitivities, growth patterns and/or migratory potential(s), as shown by Godet et al. [21,22]. Therefore, in order to best therapeutically control tumor growth and metastasis, it may be mechanistically useful also to understand the effect(s) of therapy on these cell sub-populations, so as to ultimately design therapeutic approaches selectively for those types of cancer cells that are most difficult to treat.

Using an orthotopic TNBC mouse model, that developed spontaneous metastases, Stras et al. [23,24] have previously shown that the rate and pattern of cells’ exposure to CDDP inhibits at different extents the primary tumor growth and the onset of metastasis. In particular, for the delivery of CDDP, they utilized liposomes with different combinations of two key properties, which essentially affected the levels and rate of exposure of the cancer cells to CDDP that was delivered. They included, first, the release property of CDDP from liposomes when in the tumor interstitium, to increase the CDDP penetration in the tumor; and second, the property of adhesion of liposomes to the tumors’ ECM for a slower liposome clearance from the tumors. The systematic interrogation of the combinations of these properties’ on liposomes demonstrated that the fastest, most uniform and more prolonged exposure of cancer cells to the delivered chemotherapy (by liposomes with both properties) were most effective in decreasing the tumors’ growth rate.

In this study, we aimed to interrogate the role of the rate (and pattern) of exposure of the cancer cells to CDDP on affecting (1) the extent of killing different subpopulations of cancer cells (hypoxic vs. normoxic) and/or (2) the inhibition of the cells’ motility, which may impact their metastatic potential. The present study was designed to systematically interrogate these factors on TNBC multicellular spheroids used as surrogates of the solid tumors’ avascular regions.

## 2. Materials and Methods

The lipids 1,2-diarachidoyl-sn-glycero-3-phosphocholine (20PC), 1,2-dipalmitoyl-sn-glycero-3-phospho-L-serine (sodium salt) (DPPS), 1,2-distearoyl-sn-glycero-3-phosphoethanolamine-N-[amino(polyethylene glycol)-2000]-(ammonium salt) (DSPE-PEG(2000)), 1,2-dipalmitoyl-snglycero-3-phosphoethanolamine−N-(lissamine rhodamine B sulfonyl) (ammonium salt) (DPPE-rhodamine), and the custom synthesized 1,2-distearoyl-sn-glycero-3-phosphoethanolamine-N-PEG2000-dimenthylammonium propane (DSPE-PEG(2000)-DAP, the ‘adhesion lipid’ as previously reported) were purchased from Avanti Polar lipids (Alabaster, AL, USA). Cholesterol, CDDP, and all buffers were purchased from Sigma Aldrich (St. Louis, MO, USA) and cell-related media reagents were purchased from Gibco (Waltham, MA, USA).

### 2.1. Cell Culture

The MDA-MB-231 TNBC cell line was obtained from the American Type Culture Collection (ATCC, Rockville, MD, USA) and cultured in Dulbecco’s modified Eagle’s media (DMEM) supplemented with 10% fetal bovine serum (FBS) and 100 units/mL penicillin, 100 mg/mL streptomycin at 37 °C with 5% CO_2_. The MDA-MB-231 hypoxia reporter cells (MDA-MB-231HR) were generated, as previously described in Godet et al., 2019 [21]. Briefly, the vectors encoding CMV-loxp-DsRed-loxp-eGFP (Addgene #141148) and 4xHRE-MinTK-CRE-ODD (Addgene #141147) were developed by using Gateway and InFusion cloning strategies, respectively. The final lentiviral vectors were transduced into the MDA-MB-231 cells that were selected and single-cell cloned. The selection and screening process yielded a cell-line that expresses DsRed in normoxic conditions and GFP after the prolonged exposure to hypoxic conditions. The cells were cultured in 20% and 1% O_2_ for normoxic and hypoxic conditions, respectively. Hypoxic conditions were achieved by using an InvivO_2_ hypoxia workstation (Baker, Sanford, ME, USA) with an ICONIC (Baker, Sanford, ME, USA) electronically controlled gas-mixing system maintained at 37  °C and 75% humidity, equilibrated at 1% O_2_, 5% CO_2_, and 94% N_2_.

### 2.2. NanoParticle (NP) Preparation and CDDP Loading

The compositions of the lipid NPs used in the study were either non-pH responsive or pH-responsive, as previously described in detail [23,24]. The non-pH responsive lipid NP comprised 20PC: cholesterol: DSPE-PEG (2000) at mole ratio 0.72:0.18:0.09. The pH-responsive NP were composed of 20PC: DPPS: cholesterol: DSPE-PEG-DAP at mole ratio 0.60:0.26:0.04:0.09. The pH-responsive release property of the lipid NP was enabled by a lipid membrane comprising a phosphatidylcholine lipid and a titratable anionic lipid, phosphatidylserine, with at least two carbon atoms mismatch in their acyl tail lengths. We have previously demonstrated [25,26] that this lipid bilayer is relatively well mixed at a physiological pH, due to the electrostatic repulsion among the negatively charged phosphatidyl serine lipid headgroups, resulting in the stable retention of the contents by the NP. At acidic pH values, the protonation of the phosphatidyl serine lipid headgroup and the inherent hydrogen bonding between these lipids, results in the attraction of the protonated phosphatidyl serine lipids, while they laterally diffuse on the plane of the lipid bilayer, causing the lipid phase separation and the formation of phosphatidyl serine-rich lipid domains. We have demonstrated that, at the domain boundaries, the transient lipid packing defects spanning the lipid bilayer, due to the difference in the lipid acyl tails and the different intrinsic lipid tilts relative to the plane of the bilayer, result in the increased membrane permeability and the content release from the NP. The property of a pH-triggered adhesion to the tumor ECM was enabled by adding the titratable group dimenthylammonium propane (DAP) at the free end of the PEG chains. The moiety DAP has a pKa of approximately 6.8 [27], which is comparable to the pH in the tumor interstitum [28,29]. Upon the DAP protonation, the cationic charges on the freely undulating PEG chains were shown to not result in significant interactions of the NP with cells, but instead to enable the NP to retain a certain level of adhesion to the negatively charged tumor ECM; this translated into a greater tumor uptake and a slower NP clearance from the tumors in vivo [24]. All lipid nanoparticle compositions were labeled with 0.06 mole % DPPE-rhodamine.

The lipid NPs were prepared using a thin film hydration method [23]. Briefly, a dry lipid film (10−40 μmol total lipid) was hydrated with 1 mL of phosphate-buffered saline (PBS) (10 mM phosphate-buffered saline with 1 mM ethylenediaminetetraacetic acid (EDTA), pH 7.4), and the suspension was annealed at 66 °C for 2 h following the extrusion for 21 times through 100 nm pore-size polycarbonate membranes at 80 °C (10–15 °C above the highest value of the constituent lipid transition temperature). The lipid NPs were passed through a Sepharose 4B column before CDDP was then passively loaded by adding CDDP (17 mg/mL) to the lipid NP suspension (20 mM total lipid) at 80 °C for 4 h under constant mixing. Unencapsulated CDDP was removed from the lipid NP using a Sephadex G50 column, eluted with PBS at pH 7.4. The CDDP content in the lipid NP was analyzed by lysing 100 μL of the NP suspension diluted with 300 μL of 10% HCl with 0.5% Triton X-100, followed by measuring the platinum content using a graphite furnace atomic absorption spectrophotometer (GFAAS) (using a hollow cathode Pt 365.9 nm lamp, Buck Scientific, Norwalk, CT, USA), and was quantified by the comparison of the measured signal to a calibration curve as reported in the supplemental information (Figure S1). Prior to the incubation with the cells, all lipid NPs were filter sterilized (200 μm filters, VWR, Radnor, PA, USA).

### 2.3. Characterization of the Lipid NP: ζ-Potential

The size and ζ-potential of the lipid NP were determined using a Zetasizer Nano ZS 90 (Malvern, UK). The samples were diluted in PBS (10 mM phosphate buffer, 150 mM NaCl, 300 mOsm) for sizing and/or low-salt PBS (10 mM phosphate buffer, 15 mM NaCl, 275 mM sucrose, 300 mOsm) for measuring changes in the ζ-potential as a function of pH.

### 2.4. Characterization of the Lipid NP: Release of CDDP from the NP

To evaluate the retention of CDDP by the NP, 200 µL of the NP suspension was added in 800 µL of a FBS-supplemented cell culture media (10% FBS final concentration), in the presence of cells, in the respective conditions. At the end of the incubation, the released CDDP from the NP was measured by adding 1 mL of the suspension in a Sephadex G50 column, and by collecting the eluted separate fractions that corresponded to the NP and the released CDDP. The content of platinum in the collected fractions was quantified using the GFAAS by diluting 25 μL of the sample with 75 μL of 10% HCl and adding 5 μL Triton-X. The Pt content in these samples was compared to the Pt content at the start of the incubation to determine the % CDDP retained in the lipid NP.

### 2.5. Determination of the CDDP IC_50_ Values on the Cell Monolayers

The cells were plated on 96-well plates at a density of 20,000 cells/well in normoxic and in hypoxic conditions. For the hypoxic conditions, the cells were pre-conditioned to the hypoxic environment for 48 h pre CDDP treatment. Following the required exposure, independent of the condition, the cells were exposed to free or NP containing CDDP for 6 h. For the hypoxic conditions, the experiment was performed at the naturally lowered pH 6.8 whereas for the normoxic conditions, the experiment was performed at pH 7.4 and at pH 6.8, to decouple the effects of pH and hypoxia. Following the completion of the incubation, the cells were washed twice with sterile PBS at the same pH and fresh media was added to the cells. Following two doubling times (2 × 36 h, 2 × 42 h and/or 2 × 41 h, for the cells in the normoxic conditions at pH 7.4 and 6.8, and for the cells in the hypoxic conditions, respectively), the media was removed, washed 1× with PBS and the MTT assay (Promega, Madison, WI, USA) was performed (according to manufacturer’s protocol) to assess the cell viability. The cell viability was normalized by the viability of the cells that did not receive any CDDP at the respective oxygen conditions and pH, in triplicate measurements.

### 2.6. Measurement of the CDDP Cell Uptake

In 6-well plates, 300,000 cells were plated at both normoxic and hypoxic conditions. The cells were exposed to the hypoxic conditions for 48 h prior to the CDDP introduction. The cells were incubated with 200 and 100 μg/mL of CDDP at the respective pH values and oxygen conditions. At different time points, the cells were washed twice with PBS to remove the extracellular CDDP. The cells were then carefully scraped and collected in 1 mL DDI and sonicated for 10 min before measuring the Pt content on a GFAAS, in triplicate measurements.

### 2.7. Spheroid Formation and the Cell Population Characterization

Spheroids, used as surrogates of the tumors’ avascular regions, were formed, as previously reported [23]. Briefly, the MDA-MB-231-HR cell suspension was kept on ice and Matrigel^TM^ (Corning Inc., Corning, NY, USA) and was added at a concentration of 2.5% (*v*/*v*) to promote the spheroid formation. The cells were seeded on U-shaped 96-well plates treated with poly-HEMA (Sigma Aldrich, St. Louis, MO, USA) and centrifuged at 1000 RCF for 10 min at 4 °C.

To image the spatiotemporal distributions of the cells in spheroids, a Leica LSM 780 was used at 488 nm and 514 nm excitation lasers for imaging for the GFP (508–568 nm, detecting hypoxic/post-hypoxic cells) and the RFP (578–660 nm, detecting cells experienced only normoxic conditions), respectively, using 10 µm z-stack optical slices. The spheroids were transferred to a glass bottom 35 mm cell culture dish (VWR, Radnor, PA, USA) and imaged every two days. To quantify the population distributions of the cells in the spheroids of different sizes, first, the spheroids were plated on adherent wells until they reached confluence, and then the cells were lightly trypsinized, washed, and resuspended in ice cold media before they were analyzed using a BD FACS Canto (Franklin Lakes, NJ, USA).

### 2.8. Spatiotemporal Profiles of the NPs and the Drug Surrogate in the Spheroids

To measure the spatiotemporal distributions of the NPs and of the drug surrogate in the spheroids, the NPs containing 1 mol % DPPE-rhodamine lipid were prepared and loaded with carboxyfluorescein diacetate succinimidyl ester (CFDA-SE) (ex/em: 497/517 nm), which was used as the drug surrogate. The final CFDA-SE concentration in the NP suspension was 200 nM. The extent of the retention, as a function of pH, of CFDA-SE from both types of NPs matched the extent of retention of CDDP from the corresponding NPs (see supporting information, Table S1).

The spheroids were incubated with different concentrations of free CFDA-SE and/or with 1 mM (total lipid concentration) of the lipid NP (containing CFDA-SE) for up to 6 h (to observe the ‘uptake’ into the spheroids) and then the spheroids were transferred in fresh media (to observe the ‘clearance’ from the spheroids). At different time points, the spheroids were fished out in 2 μL media and frozen in Cryochrome™gel over dry ice. The spheroids that were not incubated with the NP and/or CFDA-SE were used as background. The equatorial, 20 μm thick spheroid sections were imaged on the Leica LSM 780 microscope. The calibration curves were obtained by imaging, with the same microscope, with the known concentrations of each fluorophore in a quartz cuvette of 20 μm pathlength. At different time points, an in-house eroding code was applied to quantify the radial distributions (using 5 µm concentric rings) of the fluorescence intensities as previously reported [23]. The radially time-integrated profiles were obtained using the trapezoidal rule across the sampled time-points; n = 4–5 spheroid slices were analyzed per time point.

### 2.9. Spheroid Treatment

The spheroids of approximately 400 μm in diameter were treated with free CDDP and with CDDP delivered by the NP (NP-CDDP) for time periods scaled to their corresponding blood clearance times in mice [30,31], at CDDP concentrations proportional to the corresponding MTD values in mice. Upon completion of the incubation, the spheroids were transferred into fresh media, their size was monitored—by imaging—for 14 days, at which point the size of the non-treated spheroids reached an asymptote, and then each spheroid was plated in a single well on 96-well plates and 6-well plates to evaluate the outgrowth relative to the non-treated spheroids, detailed below, and the population distributions, respectively.

For evaluating the extent of the outgrowth, that was used as a surrogate of recurrence, the following protocol was followed: after the cells from plated, the non-treated spheroids reached confluency, then the cells that were plated from all spheroids (untreated and treated) were trypsinized and counted using trypan blue. The extent of the outgrowth of each condition was defined as the ratio of the cell counts for each condition normalized by the cell counts in the non-treatment condition.

To evaluate the population distributions, the cells from the plated spheroids for each condition, after being allowed to reach confluency in the 6-well plates, were analyzed on a BD FACS Canto, as described above.

### 2.10. Cell Migration from the Spheroids Embedded in a Matrix

To study the effect of the different treatment conditions on cancer cell migration, after the 400 μm spheroids were treated with free CDDP and NP-CDDP, as described above, the spheroids were then transferred into fresh media for two doubling times (72 h). At that point, the spheroids were then embedded in 2 mg/mL type I collagen gel by modifying the procedure, as described previously by Jimenez et al. [32]. Briefly, each spheroid was fished out in 100 μL of 1:1 (*v*/*v*) complete DMEM media and reconstitution buffer over ice, and was added to the individual wells of a pre-warmed 96-well cover-slip bottom cell culture plate and was immediately transferred to a sterile incubator at 37 °C to facilitate the homogeneous polymerization of the collagen. (The reconstitution buffer was formed by a high density soluble rat-tail collagen I (354,249, Corning Inc., Corning, NY, USA) added to obtain a final collagen I concentration of 2 mg/mL at pH =7.0 that was adjusted with 1 N NaOH). The volume of 100 μL of the warm DMEM was added to each well after 3 h, and spheroids were imaged on a Nikon Ti2 transmission microscope (Minato, Japan) equipped with (GFP and RFP filters) every 20 min for 24 h.

For the different time points, the migration distance for each cell was measured on the NIS elements software (Nikon, Minato, Japan) on both the DsRed (580–680 nm) and GFP (511–561 nm) channels for n = 3 spheroids per condition.

### 2.11. Statistical Analysis

The results are reported as the arithmetic mean of n independent measurements ± the standard deviation. The significance was evaluated by one-way ANOVA, and the Student’s t test with *p*-values less than 0.05, was considered to be significant.

## 3. Results

### 3.1. Cell Characterization

Table 1 shows that the two cell lines, the MDA-MB-231 and MDA-MB-231HR (hypoxia reporter), were indistinguishable in terms of the effect of hypoxia and/or extracellular pH on the doubling times of the cells. A comparison of the first column (normoxic/neutral pH) to the second (normoxic/acidic pH) and third columns (hypoxic/naturally acidic pH) suggests that it was the pH of the extracellular environment (acidosis) and not the oxygen levels that delayed the growth rates of cells.

### 3.2. NP Characterization

Regardless of the composition, the NP had a size range of 110–130 nm post CDDP loading (Table 2). The pH-responsive NP exhibited less negative ζ-potential with a decreasing pH, due to the protonation of the DSPE-PEG (2000)-DAP moiety, in agreement with our previous reports [24]. The non-pH-responsive NP showed no significant change in the ζ-potential with the changing pH. The CDDP loading in the NP ranged from 3.5 to 3.7%. Only the pH-responsive NP showed a significant decrease in the CDDP retention at pH 6.8, due to the triggered release (75.1% vs. 94.0%). No significant difference in CDDP retention by the NP was measured at pH 6.8 in the normoxic vs. hypoxic conditions (Table S2).

### 3.3. Ic_50_ Values and the Drug Efficacy on the Cell Monolayers

Table 3 (first row) shows that the IC_50_ value of free CDDP increased in the hypoxic conditions relative to the normoxic conditions. This was attributed to the effects of the lower oxygen level and not of acidosis—the latter is naturally occurring in the cell media in the hypoxic chamber (Figure S2)—, and was based on the observation that for free CDDP, the IC_50_ values in the normoxic conditions at a neutral pH (a) and at an acidic pH (b) were not different. The non-pH responsive NP loaded with CDDP (non-responsive NP-CDDP) did not release adequate drug at the incubation conditions to reach 50% of the cell kill. Conversely, the pH-responsive NP loaded with CDDP (responsive NP-CDDP), resulted in measurable IC_50_ values that decreased with the lowering pH (in normoxic (b) and hypoxic (c) conditions), as expected, due to the release of CDDP from the NP in the extracellular media [23,24]. Both NP types used in this study were previously shown to not significantly associate with or become internalized by the cancer cells. The strategy of the drug delivery with these NPs includes the release of free CDDP in the extracellular medium; the delivery strategy is that, ultimately, these NPs are utilized as carriers that deliver their therapeutic cargo in the solid tumor interstitium, where the released, highly diffusing drug may penetrate the tumor, resulting in uniform drug microdistributions and better tumor killing [24,33]. The resistance factor due to hypoxia (shown in column (c)) [34], defined as the ratio of the IC_50_ values in the hypoxic conditions over the IC_50_ values in the normoxic conditions at the same pH, was significant and equal to 2.6, both for the free CDDP and for the responsive NP-CDDP.

Both the MDA-MB-231 cell line and the hypoxia reporter derivative, MDA-MB-231HR, exhibited the same IC_50_ value to free CDDP (at pH 7.4, also shown in Figure S3), and, therefore, were considered equivalent for the purpose of this study.

**Figure 1:**
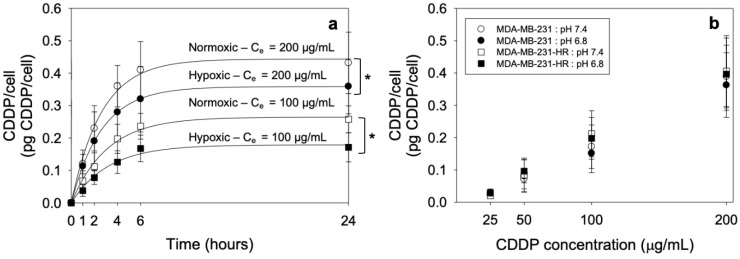
(**a**) Kinetics of the CDDP uptake in the hypoxic (black symbols) and normoxic (white symbols) conditions for 200 µg/mL (circles) and 100 µg/mL (squares) initial free CDDP extracellular concentrations. A single exponential asymptotic two-parameter growth equation was fit to obtain the rate and extent of the uptake. Values reported as mean ± standard deviation of n = 3 independent runs as shown on Table 4. * *p*-value < 0.05. (**b**) Comparison of the cell-uptake of CDDP for the MDA-MB-231 (circles) and MDA-MB-231HR cells (squares) after 6 h of incubation for the initial extracellular concentrations (Ce) of 25, 50, 100 and 200 µg/mL at pH 7.4 (white symbols) and pH 6.8 (black symbols). Values reported as mean ± standard deviation of n = 3 independent runs.

To investigate a potential mechanistic explanation for the observed reduced drug killing in the hypoxic conditions relative to the normoxic conditions, the uptake of free CDDP by the cells was measured in the corresponding conditions (Figure 1). Although the rate of the CDDP uptake remained independent of oxygen levels and/or of the initial extracellular concentration of the drug (Ce) (Table 4), the extent of the drug uptake was lowered in the hypoxic conditions relative to the normoxic conditions (Figure 1a). The extent (and rate, Figure S4) of the free CDDP uptake was not different between the MDA-MB-231 cells and the MDA-MB-231HR cells at both pH 7.4 and 6.8, in the normoxic conditions (Figure 1b).

### 3.4. Spheroid Characterization

**Figure 2:**
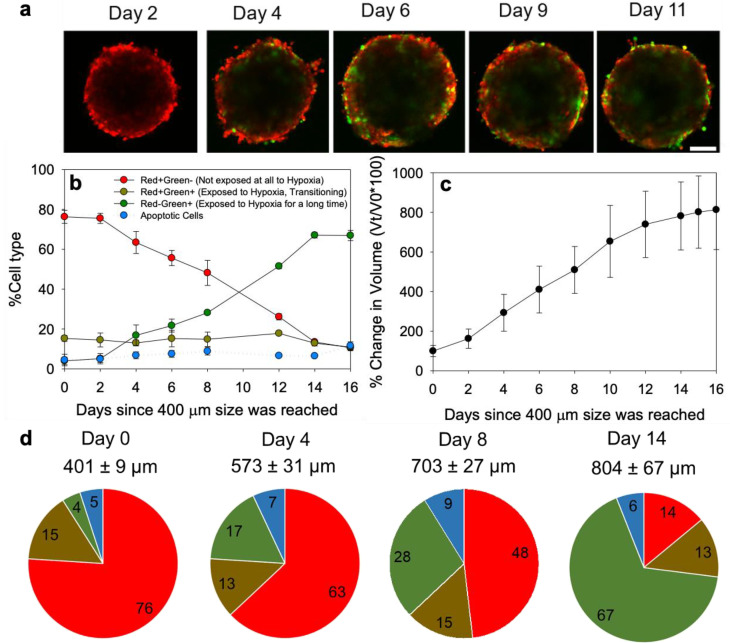
(**a**) Representative confocal microscopy images of the MDA-MB-231HR spheroids, acquired at the longest possible optical depth, where the hypoxic/post-hypoxic cells (green, GFP channel) and the cells that only experienced normoxia (red, in RFP channel) were imaged simultaneously. The optical slices shown were acquired at the spheroids’ equator up to the spheroid sizes of approximately 400 µm in diameter. For the larger than 400 µm in diameter spheroids, the optical slices shown indicate the longest working distance within the spheroids at which the photon counts were adequate. Scale bar corresponds to 200 µm. (**b**) Distributions of the cell populations in the spheroids, as measured by flow cytometry, and (**c**) the increase in the spheroid size, over time. (**d**) Pie chart representation of the MDA-MB-231HR TNBC cell populations in the spheroids of different sizes: cells that have not experienced hypoxia (red), cells that were transitioning or partially exposed to the hypoxic conditions (brown), cells that have experienced hypoxic conditions (green) and cells that have undergone apoptosis (cyan) were tracked over time, using flow cytometry, and were plotted as a percentage of the total cell population. Values reported as mean ± standard deviation of n = 6 independent spheroids, per time point.

Confocal images of the spheroids formed by the MDA-MB-231HR cells demonstrated heterogeneous spatial distributions of the cells that have experienced hypoxic conditions (green) and the cells that have only experienced normoxic conditions (red) (Figure 2a). As the size of the MDA-MB-231HR spheroids grew from 400 μm to 800 μm in diameter, the cell population distributions evolved. The population of the cells that have experienced hypoxic conditions (Figure 2b, green symbols) grew monotonically after day 4 (spheroid diameter > 573 ± 31 µm, Figure 2c) whereas the population of the cells that never experienced hypoxic conditions decreased (Figure 2, red symbols). The population distribution was unchanged post day 14 (spheroid diameter > 804 ± 67 µm) with >65% of the cells having had experienced hypoxic conditions within the spheroids (green symbols). The flow cytometry results for the selected days are shown in Figure 2d. The observed changes in the population distributions (increasing fraction of hypoxic cells) could be attributed to the increasing formation of the hypoxic regions within the spheroids of increasing size; the relatively slow O_2_ transport within the spheroids’ interstitium is expected to have contributed to the generation of the heterogeneous O_2_ spatiotemporal microdistributions which would become more pronounced as the spheroid size increased over time. One of the reasons of the development of hypoxic and/or of acidic microenvironments within the spheroids (the acidity was demonstrated before, Figure S12 in [23]), is due to the limited diffusivities of oxygen and protons in the spheroid interstitium, relative to the rates of their consumption or production, respectively, by the cells comprising the spheroids.

### 3.5. Microdistributions in the Spheroids of a Drug Surrogate for the Different Delivery Approaches

**Figure 3:**
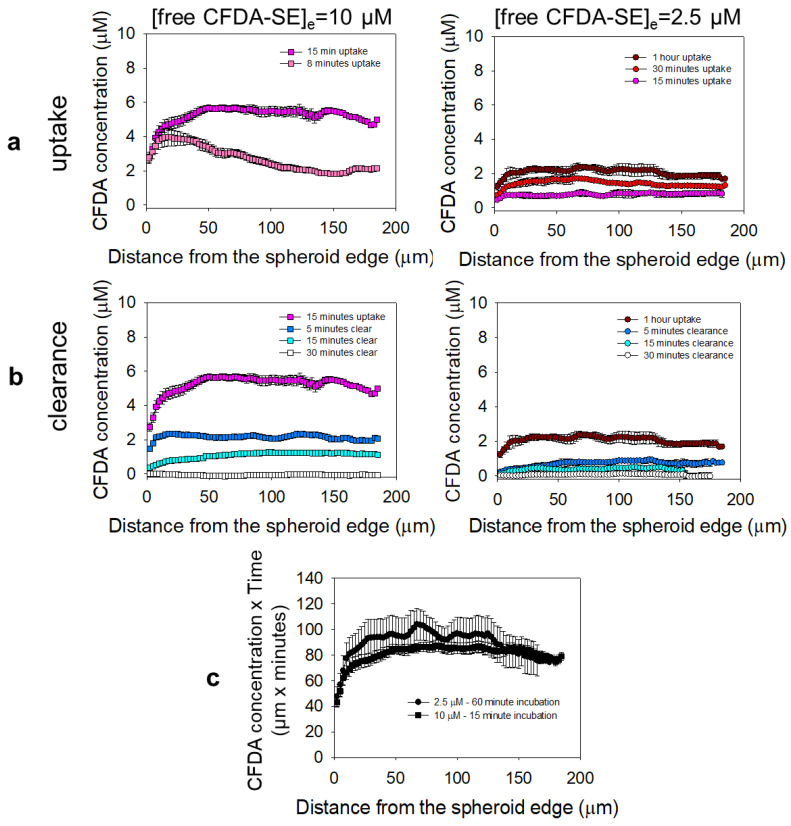
Different rates of exposure at the same ‘cumulative dose’ of a surrogate of the free drug in the spheroids. Microdistributions in the spheroids of the fluorophore CFDA-SE that was incubated with the spheroids in free form at 10 µM (left column, for up to 15 min) and 2.5 µM (right column, for up to 1 h), and was used as the drug surrogate. (**a**) “Uptake” microdistributions over time, when the spheroids were incubated with the free CFDA-SE in the surrounding media. (**b**) “Clearance” microdistributions over time, when the spheroids were transferred in media without CFDA-SE. (**c**) The time-integrated concentrations of the drug surrogate (AUC) along the spheroid radius for the corresponding incubation conditions indicate similar profiles (cumulative radial doses) within the spheroids. Values reported as mean ± standard deviation of n = 3–4 spheroids imaged per time point.

In an effort to ultimately compare the effect of the rate of the drug delivered to the cells on cell killing, while keeping the same cumulative delivered dose, we measured and compared the radially integrated spatiotemporal distributions (the area under the curve, AUC) of a drug surrogate (free CFDA-SE) within the spheroids (Figure 3a–c). Figure 3c shows that the AUCs of the free CFDA-SE within the spheroids were identical when the concentration and time of incubation of the free CFDA-SE in the media were adjusted to result in equal products (10 µM × 15 min = 2.5 µM × 60 min).

**Figure 4:**
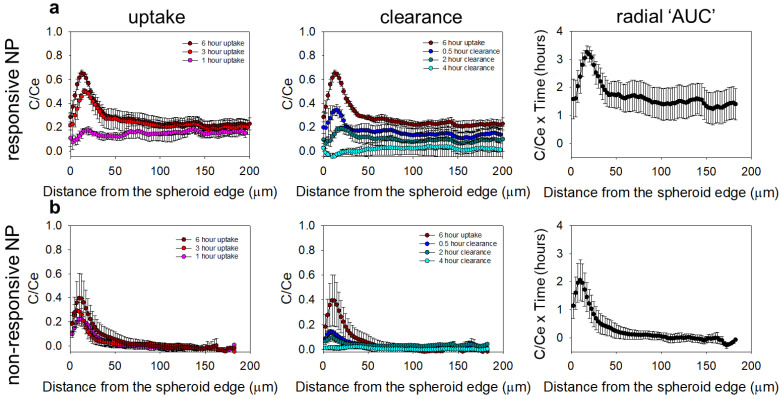
Microdistributions in the spheroids of the fluorophore CFDA-SE delivered by (**a**) responsive NP, and (**b**) non-responsive NP. The first column shows the spatiotemporal microdistributions during the “uptake” (in the presence of the NP), and the second column during the “clearance” (after the removal of the NP from the incubation medium) of the delivered CFDA-SE in the spheroids. The plots on the righthand column show the time-integrated concentrations of the drug surrogate (AUC) along the spheroid radius for the corresponding delivery approaches. Values reported as mean ± standard deviation of n = 3–4 spheroids per time point.

When the drug surrogate was delivered by the NP, in agreement with previous studies in spheroids [24]—which were shown to capture the diffusion-limited transport in tumor avascular regions—the lipid NP with both pH-responsive properties (i.e., the ECM-adhesion and the interstitial release of contents) shown in Figure 4a, resulted in a greater penetration of the delivered drug surrogate (CFDA-SE fluorophore) and greater values of the time-integrated concentrations (AUC) of the delivered fluorophore, than the extent of penetration and level of the AUC of the same fluorophore when delivered by the NP without these properties (Figure 4b).

### 3.6. Effect of the Rate of the Free CDDP Treatment on the Spheroids: Cell Survival

**Figure 5:**
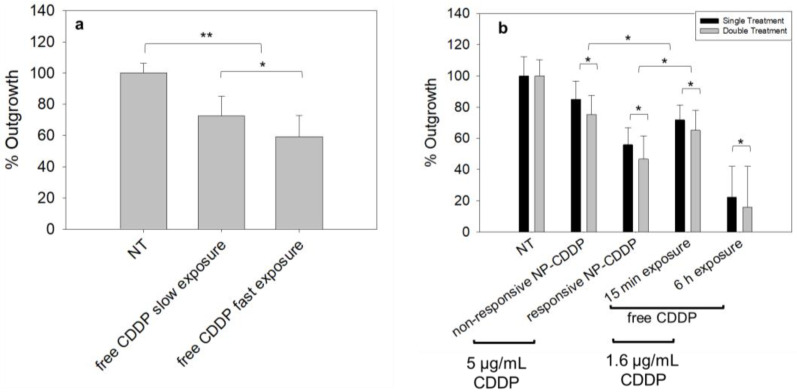
Effect of the CDDP treatment regimen on the extent of the cell outgrowth from the MDA-MB-231HR spheroids. (**a**) Effect of the rate of the spheroid exposure to free CDDP at the same total cumulative dose (demonstrated in Figure 3c). Free CDDP/slow exposure corresponded to 60 min spheroid exposure to 0.4 µg/mL free CDDP. Free CDDP/fast exposure corresponded to 15 min spheroid exposure to 1.6 µg/mL free CDDP. (**b**) In treatment studies of spheroids with NP-CDDP, CDDP concentrations in the incubating medium of 5 µg/mL for a single treatment, and 2.5 µg/mL for a double treatment, followed by 1 µg/mL free CDDP, as shown on Table S3. Percentage outgrowth relative to the non-treatment was calculated as follows: following the exposure to therapy, the spheroid size was monitored until the non-treated spheroids stopped growing in size (14 days post treatment). At that point, each spheroid was plated on a separate adherent well, and when the non-treated condition reached confluency, the number of cells from each treatment were counted and normalized to the number of cells from the non-treated cases. For each treatment, n = 18 spheroids were evaluated across n = 3 independent preparations. *p*-values: * < 0.05, ** < 0.01.

To evaluate the effect of the rate of free CDDP delivered to the cells on cell killing, for the same cumulative delivered dose, the spheroids were incubated with free CDDP (at high, 1.6 µg/mL, and low, 0.4 µg/mL, concentrations) for short (15 min) and long (60 min) periods, respectively. The two treatments of free CDDP were expected to have comparable AUCs within the spheroids, based on the findings of the fluorescent drug surrogate CFDA-SE, shown in Figure 3c. Figure 5a shows that the faster rate of delivery of free CDDP resulted in a significantly better inhibition of the cell growth compared to the same cumulative dose of CDDP that was delivered at a slower rate.

### 3.7. Effect of the CDDP Delivery Regimens on the Spheroids: Cell Survival

**Figure 6:**
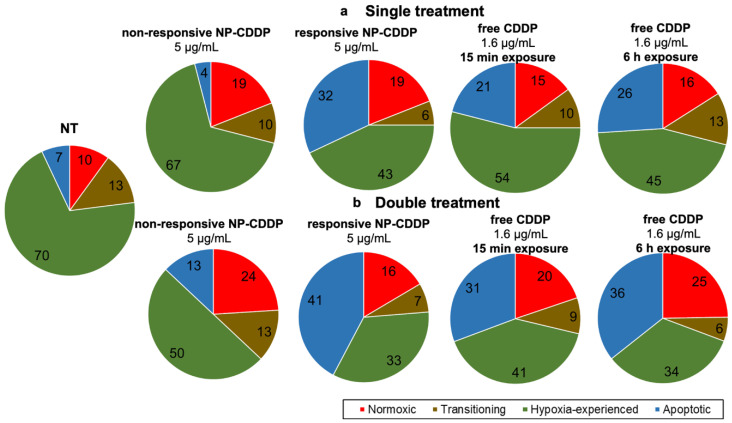
Effect of the type of the CDDP treatment(s) on the progression of the cell populations in the spheroids, evaluated using flow cytometry. Percentage of the cell population of the cells for single (**a**) and double (**b**) treatment with CDDP in different forms. Cells that have not experienced hypoxia (red), cells that were transitioning or partially exposed to hypoxic conditions (brown), cells that have experienced hypoxic conditions (green), and cells that have undergone apoptosis (blue). CDDP concentration was 5 µg/mL in the single treatments, and 2.5 µg/mL in the double treatments (followed by the same dose of free CDDP, as summarized on Table S3). Charts represent the averaged values of n = 3 spheroids per treatment for n = 3 independent treatment preparations/ measurements.

To compare the cell killing efficacy, in the spheroids, of the NP-delivered CDDP and of the free CDDP, the therapeutic treatments were scaled according to the corresponding pharmacokinetic time scales and the reported maximum tolerated doses in mice (MTD). Pharmacokinetically, free CDDP is reported to clear 20–27 times [31] faster than the lipid NP; hence, the spheroids were incubated with free CDDP for 15 min and with the NP-CDDP for 6 h. Regarding the incubation concentrations of CDDP in free vs. NP forms, free CDDP was reported to exhibit MTD values 2.7–3.2 times lower than the MTD values of the liposomal/NP- CDDP [35]. Therefore, the spheroids were incubated with 5 µg/mL and 1.6 µg/mL of CDDP in NP and free form, respectively. In addition, two dosing schedules were designed to compare the efficacy and the population distributions in the spheroids post treatment, as shown in Figure 5b and Figure 6 and Table S3. First, in the single treatment, all of the drug was delivered at day 0, and the incubation time was scaled to the blood clearance kinetics of the respective carrier. Second, in the double treatment, only half of the dose was delivered at day 0, and then a reduced dose of free CDDP was delivered on day seven on all conditions. Overall, the double treatment involved significantly less total concentrations of CDDP (ranging from 20 to 30 % less, compared to the single treatment). The 6 h treatment with free CDDP was used as a reference of the maximum possible achievable killing effect in vitro, without an in vivo analogue, since free CDDP infused for 6 h at this relative concentration would not be expected to be tolerated well.

Following a single treatment with CDDP (Figure 5b, black bars), the greatest suppression of the cell outgrowth was observed when CDDP was delivered by the responsive NP-CDDP, followed by the treatment with the free agent (for a 15 min incubation), in agreement with previous reports [24]. This order in efficacy was attributed to the corresponding microdistributions of CDDP in the spheroid, and followed the trend of the AUC plots shown in Figure 3 and Figure 4. In the double treatment regimens (Figure 5b, gray bars), the trend in efficacy was unchanged. Importantly, the therapeutic effect was similar to the effect for the single treatment, although the cumulative concentration of CDDP that was incubated with the spheroids was significantly less than the CDDP concentration used in the single treatment approach.

### 3.8. Effect of the CDDP Delivery Regimens on the Spheroids: Cell Population Progression

Figure 6 shows that the non-treated spheroids had the highest fraction of the hypoxia experienced cells (green), possibly attributed to the greater size of these spheroids over the time-frame of the experiment (as also indicated by Figure 2). When CDDP was delivered by the pH-responsive lipid NP (responsive NP-CDDP) it resulted in the greatest reduction of the hypoxia experienced cells, both after single and double treatments; it also resulted in the maximum apoptotic cell population when compared to other treatments. The double treatment, using the responsive NP-CDDP, not only managed to further lower the overall % outgrowth (Figure 6b) but also further reduced the population of the cells that were hardest to kill (cells in hypoxic conditions shown in green; Figure 6b). Figure S5 shows examples of flow cytometry plots for each treatment.

### 3.9. Effect of the CDDP Delivery Regimens on the Spheroids: Cell Migration

**Figure 7:**
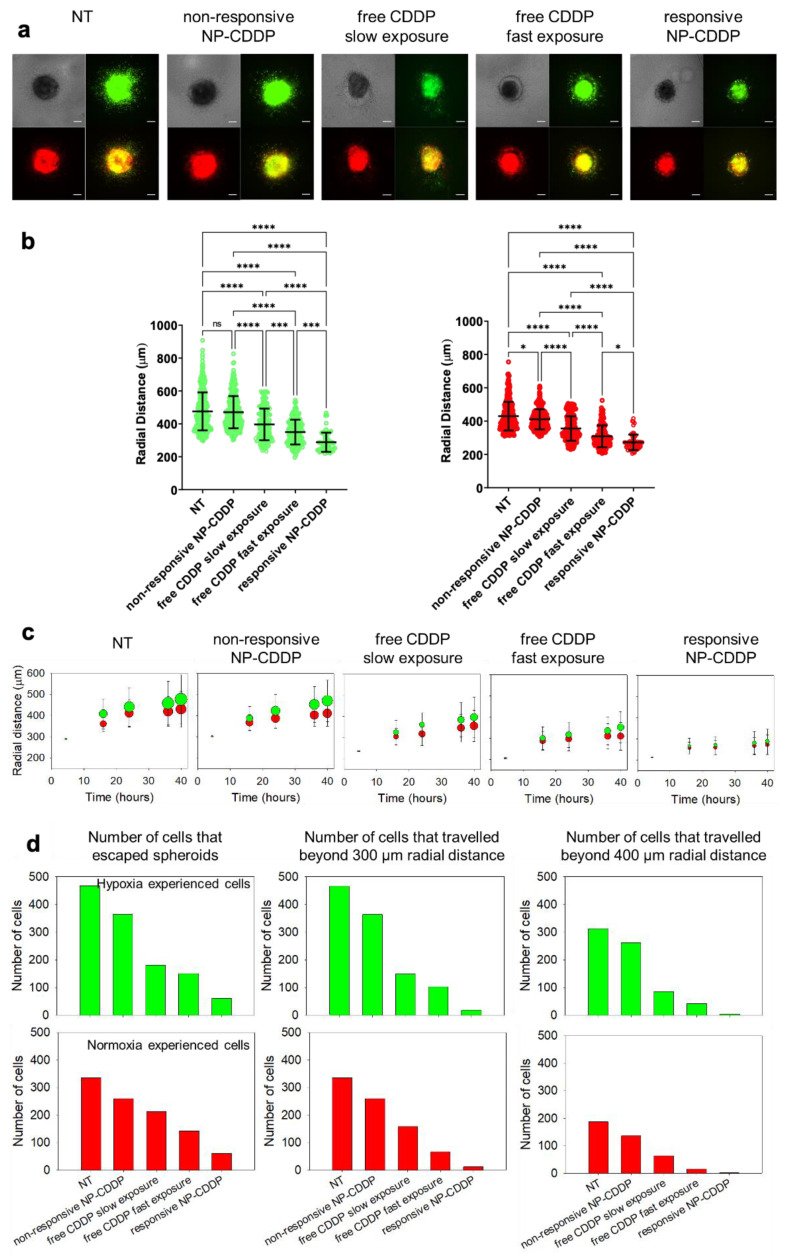
Effect of the different CDDP treatments on the migration of cells from the spheroids. (**a**) Images of the representative spheroids after 40 h post embedment in collagen, following different treatments with CDDP. Images are shown in brightfield, in the GFP (green) channel (showing cells having experienced hypoxic conditions) and the RFP (red) channel (showing cells in the normoxic conditions), and merged. Scale bar corresponds to 200 µm. (**b**) Radial locations of the cells that migrated, from the spheroid into the collagen gel, relative to the spheroid center (migration distance), at the same time point of 40 h as shown in (**a**). Cells in the normoxic conditions are shown in red, and the cells that have experienced hypoxic conditions in green. Data collected from n = 3 spheroids per treatment. Horizonal lines indicate the mean and standard deviation averaged over the entire cell counts. *p*-values: * < 0.05, *** < 0.001, **** < 0.0001. (**c**) Average migration distances over time for the two cell populations: cells in the normoxic conditions (red), and cells that have experienced hypoxic conditions (green). The symbols’ area was scaled to the number of cells for each condition. The reported distances correspond to the mean and standard deviation of the distances of all cells migrated from n = 3 spheroids, and quantified as explained in (**b**). (**d**) Number of cells at different distances from the spheroid center at t = 40 h post spheroid embedment in collagen. Free CDDP/slow exposure corresponded to the 60 min spheroid exposure to 0.4 µg/mL free CDDP. Free CDDP/fast exposure corresponded to the 15 min spheroid exposure to 1.6 µg/mL free CDDP. In treatment studies of the spheroids with NP-CDDP, CDDP concentrations in the incubating medium, was 5 µg/mL for a 6 h exposure.

Figure 7 shows that the rate of exposure to CDDP of the cells in the MDA-MB-231HR spheroids affected not only the overall cell survival (as shown in Figure 5) and the progression of the cell populations (as shown in Figure 6), but also the extents of the distances travelled by the cells (cell migration). For treatments with free CDDP, the faster rate of exposure (free CDDP/ fast exposure) resulted in a greater cell kill (Figure 5a) and in shorter distances (Figure 7b) travelled outside the spheroids by fewer cells (Figure 7c,d), compared to the slow rate of the exposure to free CDDP with the same AUC within the spheroids (Figure 3c). Similarly, when delivered by the pH-responsive NP (responsive NP-CDDP), the treatment resulted in the best inhibition of migration, both in terms of the distance travelled by the cells and in terms of the number of cells that travelled the longer distances. As expected [21,36], the cells that have experienced hypoxia (green) travelled longer distances than cells in the normoxic conditions (red) (Figure S6). Interestingly, the faster rate of exposure to CDDP decreased cell migration, independent of the cells being in hypoxic or normoxic environments (Figure 7b,d).

## 4. Discussion

Although CDDP has shown promising results in the treatment of various cancer types [37], its use in TNBC patients has been limited [38]. This is partly due to the serious adverse effects and the limited responses in patient subpopulations which have narrowed patient compliance [39]. Side effects may, sometimes, be addressed by the formulation of agents in particles, including the lipid nanoparticles (liposomes) [10]. However, although the lipid nanoparticle-delivered chemotherapies, such as Doxil^TM^, have been used in solid tumor treatments for over two decades, there is still not an FDA approved nanoparticle formulation of CDDP [40,41,42]. In this study (1) we investigated the effect of the rate of exposure to CDDP on the survival and on migration of TNBC cells, and, based on these findings, (2) we showed that, with an engineered lipid nanoparticle that enabled a fast exposure of the cells to CDDP, we could not only manage to better arrest tumor growth in general, but also to control these cancer cell subpopulations that usually survived post chemotherapy, delivered in a conventional manner, and to limit their migratory potential. The nanoparticles maximized both the rate of the cell exposure to CDDP and the number of cells affected by CDDP, when in the spheroids, which were used as a surrogate of the solid tumors’ avascular regions. The findings on the cell migration may have a particular significance in TNBC, which, is characterized by a greater frequency of metastases outside the breast, compared to other types of breast cancer [8].

The development of resistance to chemotherapy has long been a major clinical challenge, and a key determinant of cancer mortality. Enriching information on cancer cell resistance mechanisms to platinum compounds [9,43] enhances our knowledge and understanding of the complexity in molecular pathways affecting drug sensitivity. In addition to this aspect, the evolving intratumoral heterogeneity may also play a role in a solid tumor’s response to chemotherapeutics. In particular, we showed in vitro that in addition to transport (drug diffusion) limitations, that decrease bioavailability in the delivered chemotherapeutics, the development of hypoxia within the spheroids may trigger a chemoresistance in TNBC, as demonstrated by the 2–3 fold higher IC_50_ values of free CDDP to hypoxic cells. In agreement with previous studies on elucidating the mechanisms of cell resistance to CDDP [9], we also showed that its lowered efficacy, in the presence of hypoxia, was partly due to the lower CDDP cell uptake compared to the CDDP cell uptake in the normoxic conditions. This can be most likely attributed to HIF-1 regulating the downstream processes of apoptosis, autophagy and necrosis under hypoxia. Acidosis did not seem to affect the cell sensitivity to CDDP.

In addition to hypoxia and the diffusion-limited transport within the spheroids, we investigated the potential role of the rate of exposure of cancer cells to cisplatin on affecting their killing and/or their metastatic potential. We discovered that a faster rate of cell exposure to the same drug (of the same cumulative dose, approximated as the same AUC in the spheroids) caused: (1) more cell killing, and (2) decreased the distances travelled outside the spheroids by fewer cancer cells that survived treatment. Similar results were obtained when CDDP was delivered by the responsive NP-CDDP that were designed to not only release the agents within the tumor interstitium, where the agents may diffuse deeper and reach more cancer cells, but also were designed to bind to the tumor ECM so as to exhibit a delayed tumor clearance and, therefore, to achieve greater tumor delivered doses [24,33]. In all of our studies, the hypoxia reporter TNBC cells were key indicators demonstrating that this cell subpopulation was more difficult to kill, and travelled longer distances, compared to the cells that had only experienced normoxic conditions, in agreement with previous reports [21,22]. The studies presented herein provide a mechanistic explanation of our previous reports on the efficacy of the responsive NP-CDDP to delay the rate of growth of the spontaneous MDA-MB-231 TNBC metastases in a mouse model [24].

Previously, Petr et. al. [44], in an effort to control toxicities, have shown in the clinic that successive low doses of CDDP were non-inferior to a single high dose in inhibiting tumor growth. This was attributed to increased patient compliance as opposed to responses to high-dose regimens. In an effort to investigate treatment schedules of CDDP delivered by the NP, we compared in vitro a single treatment of TNBC spheroids to a double treatment schedule, where the total cumulative CDDP dose was almost half of the dose used in the single treatment. The latter treatment not only showed better efficacy in limiting cell viability, but also further reduced the hypoxia-experienced cell population, compared to single treatment. In the same studies, we showed that the delivery of CDDP by the pH-responsive NP had the best effect on limiting cell survival and migration. Evaluation of the double treatment with the NP in mouse models bearing hypoxia-reporting TNBC cells would be the next step to not only validate the superior efficacy of the responsive NP-CDDP but to also investigate the ability to control the hypoxic cell population. This type of ‘fractionated’ or multi-dose scheduling may ultimately contribute to lower toxicities in vivo.

## 5. Conclusions

Hypoxic regions in the tumor microenvironment contribute to a poorer prognosis due to the development of resistance to chemotherapy and/or due to the higher metastatic potential of the cells that have experienced hypoxia. Increasing the rates of cancer cell exposure to cisplatin, while keeping the same total cumulative dose, resulted in a decreased (hypoxic) cell survival and (hypoxic) cell metastatic potential. We demonstrated that the pH-responsive nanoparticles delivering cisplatin to the TNBC multicellular spheroids, used as surrogates of the tumors’ avascular regions, (1) fast and uniformly exposed all cells to cisplatin, (2) best inhibited cell survival, (3) lowered the surviving population of hypoxia-experienced cells and (4) decreased their migration potential, compared to conventional NP and to the free drug.

## Figures and Tables

**Table 1:** Doubling times (in hours) of the TNBC MDA-MB-231 and MDA-MB-231-HR (hypoxia reporter cells) in the normoxic conditions, at pH 7.4 (left column) and 6.8 (middle column), and in the hypoxic conditions (right column), shows that the two cell lines were indistinguishable. Acidosis, and not the oxygen levels, delayed the growth rates of the cells. Values reported as mean ± standard deviation of n = 3 independent runs. * *p*-value < 0.05.

	Normoxic; pH 7.4 (h) (n = 3)	Normoxic; pH 6.8 (h) (n = 3)	Hypoxic; pH 6.8 (h) (n = 3)
MDA-MB-231	36 ± 1	42 ± 2	41 ± 1
MDA-MB-231-HR (Hypoxia Reporter)	36 ± 1 *	41 ± 2 *	41 ± 2

**Table 2:** Characterization of the CDDP loaded pH-responsive and non-pH-responsive NPs used in this study. (R) stands for release and (A) for the adhesion property. Values reported as mean ± standard deviation of n = 6 independent NP preparations. *p*-values: * <0.05, ** <0.01.

	Size (nm) (n = 6)	PDI (n = 6)	% CDDP Loading (n = 6)	% Contents Retained at pH 7.4 after 6 h (n = 6)	% Contents Retained at pH 6.8 after 6 h (n = 6)	ζ-Potential (mV) (n = 3)		
						pH 7.4	pH 6.5	pH 6
non-pH-responsive NP (R-A-) (non-responsive NP-CDDP)	115.5 ± 7.1	0.06 ± 0.04	3.7 ± 0.3	93.0 ± 5.1	90.1 ± 8.1	−3.78 ± 2.63	−4.28 ± 2.68	−4.42 ± 2.43
pH-responsive NP (R+A+) (responsive NP-CDDP)	118.6 ± 3.3	0.08 ± 0.06	3.5 ± 0.4	94.0 ± 7.9 *	75.1 ± 6.9 *	−2.66 ± 0.40 **	−0.88 ± 1.03	−0.70 ± 1.20 **

**Table 3:** IC_50_ values (concentrations of CDDP in µg/mL required for achieving 50% killing of the MDA-MB-231 cells) in (a) normoxic conditions at pH 7.4, (b) normoxic conditions at pH 6.8 and (c) hypoxic conditions that naturally develop pH 6.8, incubated as free CDDP, CDDP loaded in the non-pH responsive NP (non-responsive NP-CDDP) and in the pH-responsive NP (responsive NP-CDDP). The resistance factor was evaluated as the ratio of the IC_50_ values in the hypoxic conditions to the IC_50_ values in the normoxic conditions at the same pH of 6.8. Values reported as mean ± standard deviation of n = 3 independent runs. ** *p*-value < 0.01; NA: 50% cell kill was not reached (dose-effect curves are shown in Figure S3B).

	IC_50_ Normoxic Conditions pH 7.4 (a) (n = 3)	IC_50_ Normoxic Conditions pH 6.8 (b) (n = 3)	IC_50_ Hypoxic Conditions pH 6.8 (c) (n = 3)	Resistance Factor (IC_50_ Hypoxic/IC_50_ Normoxic at pH 6.8) (c)/(b)
free CDDP	9.2 ± 1.2 (10.5 ± 1.4 for HR cells)	10.1 ± 1.6	26.8 ± 2.4	2.6 ± 0.5
non-responsive NP-CDDP	NA	NA	NA	NA
responsive NP-CDDP	230.9 ± 8.2 **	44.9 ± 7.8 **	116.7 ± 9.8 **	2.6 ± 0.5

**Table 4:** Estimated parameters of the single exponential asymptotic two-parameter growth equation fit y(t) =a ^×^ (1 − e^−bt^) that was applied to obtain the rate and extent of the uptake of free CDDP by the cells shown on Figure 1a. Values reported as mean ± standard deviation of n = 3 independent runs. *p*-value: *, *# < 0.05.

C_e_ (μg/mL)	Condition	a (μg/mL)	b (h^−1^)	ln2/b (h)
200	Normoxia	0.44 ± 0.01 *	0.38 ± 0.03	1.82 ± 0.13
	Hypoxia	0.36 ± 0.03 *	0.38 ± 0.04	1.85 ± 0.21
100	Normoxia	0.26 ± 0.01 *^#^	0.32 ± 0.02	2.16 ± 0.11
	Hypoxia	0.18 ± 0.01 *^#^	0.31 ± 0.04	2.21 ± 0.19

## Data Availability

All data are presented within the article and the Supplementary Materials.

## Supplementary Materials

The following supporting information can be downloaded at: https://www.mdpi.com/article/10.3390/pharmaceutics14102184/s1, Figure S1: Calibration curve of the platinum content using AAS; Figure S2: Extracellular pH (pHe) in the normoxic and hypoxic conditions; Figure S3: IC_50_ studies; Figure S4: Kinetics of the CDDP uptake by the cells; Figure S5: Characterization of the cell populations by flow cytometry after treatment; Figure S6: Effect of the treatment(s) on the migration of cells from the spheroids; Table S1: Extent of the retention of CFDA-SE from both types of NPs at different pH conditions; Table S2: Extent of the retention of CDDP from both types of NPs at different pH values in the normoxic and hypoxic conditions; Table S3: Concentrations of CDDP and the treatment duration for the single and double treatment schedules of the spheroids.
