# Supplementary material for: The Rate of Cisplatin Dosing Affects the Resistance and Metastatic Potential of Triple Negative Breast Cancer Cells, Independent of Hypoxia
Source: Pharmaceutics. 2022 Oct 13;14(10):2184. doi: 10.3390/pharmaceutics14102184 (PMC9611288; doi:10.3390/pharmaceutics14102184)
Supplement: Supplementary file 1 [file pharmaceutics-14-02184-s001.zip › pharmaceutics-1941854-supplementary.pdf]

pharmaceutics

Supplementary Materials: The Rate of Cisplatin Dosing Affects the Resistance and Metastatic Potential of Triple Negative Breast Cancer Cells, Independent of Hypoxia

Omkar Bhatavdekar, Inês Godet, Daniele Gilkes and Stavroula Sofou

Table S1. Extent of retention of CFDA-SE from both types of NP at different pH conditions. Values reported as mean ± standard deviation of $n = 6$ independent NP preparations. p-values: * < 0.01.

	% Retention at pH 7.4 after 6 hours	% Retention at pH 6.8 after 6 hours
non-responsive NP	98.4 ± 2.4	91.6 ± 3.7
responsive NP	91.5 ± 1.9*	69.3 ± 2.8*

Table S2. Extent of retention of CDDP from both types of NP at different pH values in normoxic and hypoxic conditions. Values reported as mean ± standard deviation of repeated measurements as indicated.

	Normoxic Conditions		Hypoxic Conditions	
	% CDDP retention at pH 6.8 after 6 hours (n=5)	% CDDP retention at pH 6.8 after 24 hours (n=3)	% CDDP retention at pH 6.8 after 6 hours (n=3)	% CDDP retention at pH 6.8 after 24 hours (n=3)
non-responsive NP-CDDP	90.1 ± 8.1%	84.2 ± 11.3%	88.4 ± 8.4%	83.8 ± 9.2%
responsive NP-CDDP	75.1 ± 6.9%	69.6 ± 7.7%	73.3 ± 7.4%	70.2 ± 5.8%

Table S3. and Schematic. Concentrations of CDDP and treatment duration for the single and double treatment schedules of spheroids.

	Modality	Day 0	Day 7	Day 14 – Spheroids plated for outgrowth
Single Treatment	non-responsive NP-CDDP	5 µg/mL		
	responsive NP-CDDP	5 µg/mL		
	[free CDDP] 15 min exposure	1.6 µg/mL		
	[free CDDP] 6 h exposure	1.6 µg/mL		
Double Treatment	non-responsive NP-CDDP	2.5 µg/mL	1 µg/mL	
	responsive NP-CDDP	2.5 µg/mL	1 µg/mL	
	[free CDDP] 15 min exposure	0.8 µg/mL	1 µg/mL	
	[free CDDP] 6 h exposure	0.8 µg/mL	1 µg/mL	

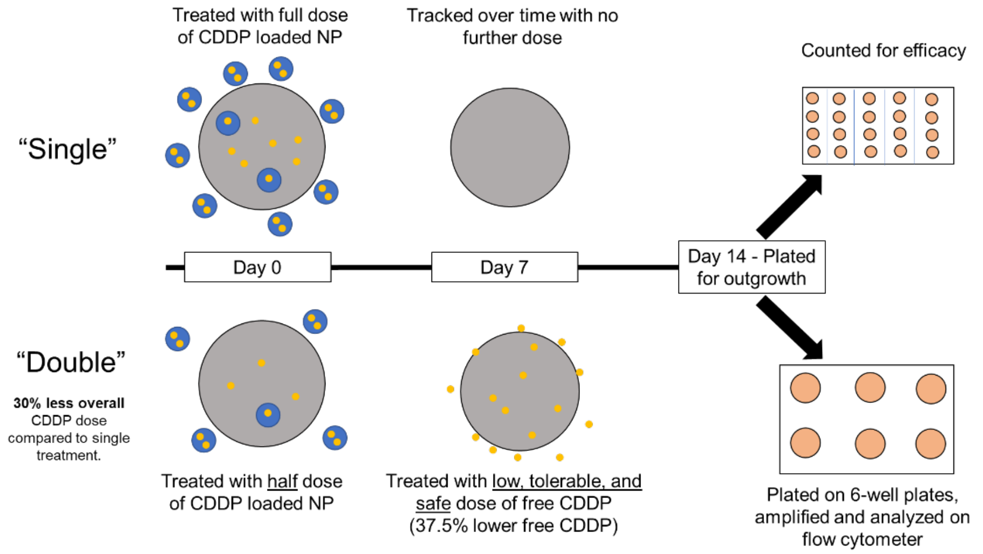

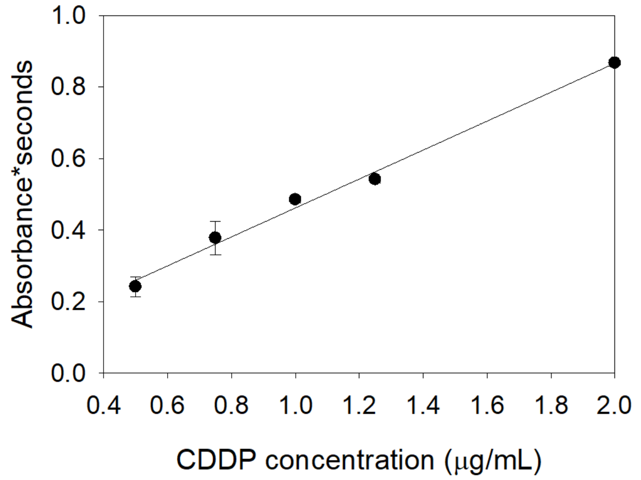

Figure S1. Calibration curve of the platinum content using AAS. Values reported as mean ± standard deviation of n = 3 independent runs.

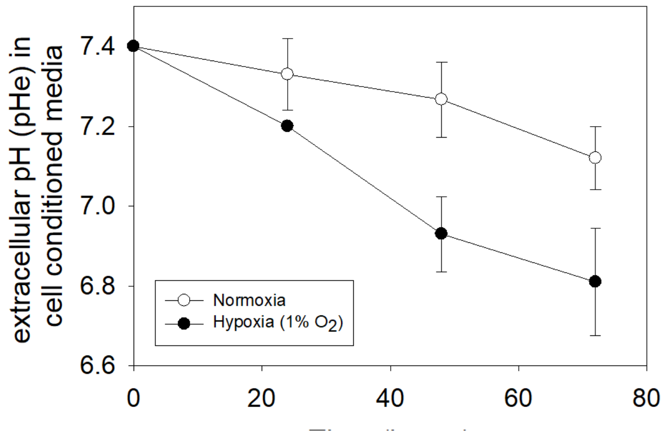

Figure S2. Extracellular pH (pHe) in normoxic and hypoxic conditions. Measurements of the extracellular pH (pHe) over time of the MDA-MB-231 cell conditioned media in normoxic (white symbols) and hypoxic (1% O_2) conditions (black symbols). Values reported as mean ± standard deviation of n = 3 independent runs.

(**A**)

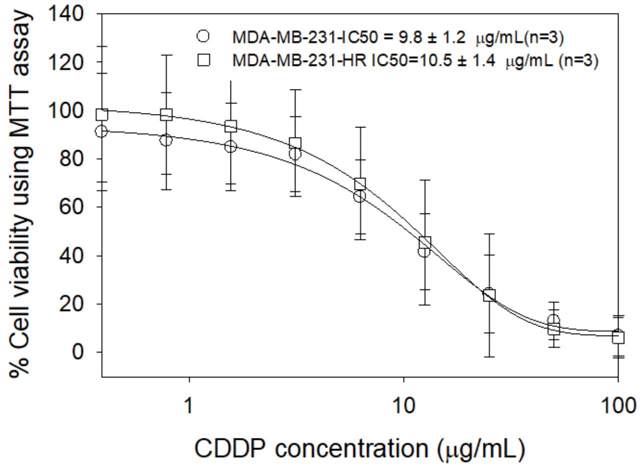

(**B**)

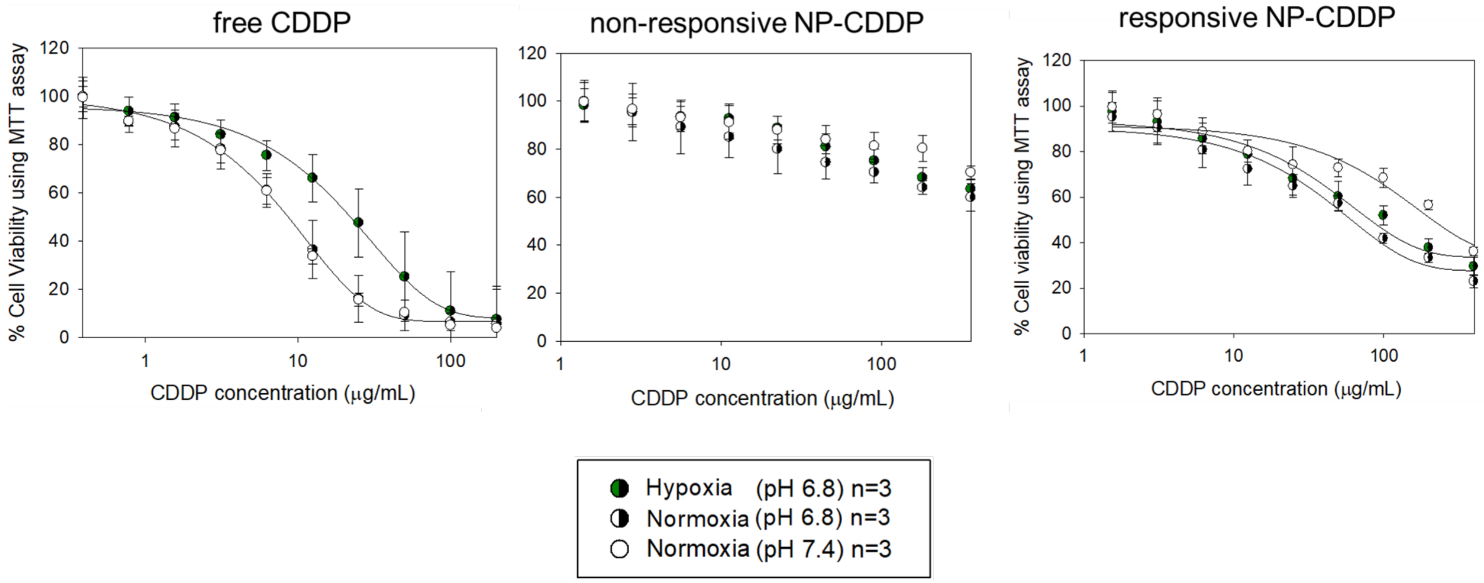

Figure S3. IC_{50} studies.

(**A**) Viability of MDA-MB-231 cells and the hypoxia reporting counterparts MDA-MB-231HR cells as a function of the extracellular concentration of free cisplatin (CDDP). Cells, in monolayers, were incubated for 6 hours with free CDDP, were then washed and incubated for two doubling times, and then a MTT assay was used to measure cell viability that was reported relative to non-treated cells. Values reported as mean ± standard deviation of $n = 3$ independent runs.

(**B**) Dose-response curves of MDA-MB-2231 cells incubated with different forms of cisplatin (CDDP) in normoxic conditions (at physiologic and acidic extracellular pH values), and in hypoxic conditions (that are accompanied by acidification of the extracellular media). Values reported as mean ± standard deviation of $n = 3$ independent runs. .

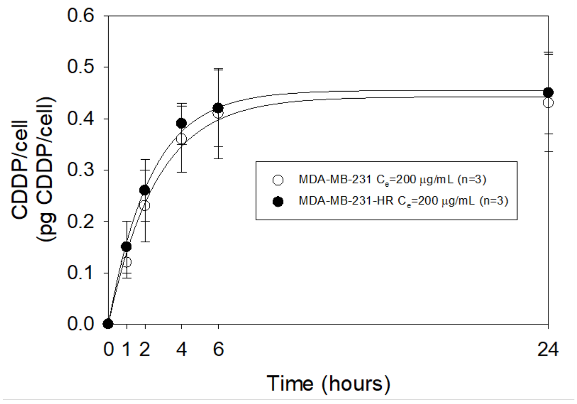

Figure S4. Kinetics of CDDP uptake by cells.

Kinetic parameters of the single exponential asymptotic two-parameter growth equation fit **$y(t) = a*(1-e^{-bt})$** that was applied to obtain the rate and extent of uptake of free CDDP by cells shown on Figure S4.

C_e (μg/mL)	Cell line	a (μg/mL)	b (hr^{-1})	ln2/b (hr)
200	MDA-MB-231	0.44 ± 0.01	0.38 ± 0.03	1.82 ± 0.13
200	MDA-MB-231-HR	0.45 ± 0.02	0.41 ± 0.04	1.68 ± 0.18

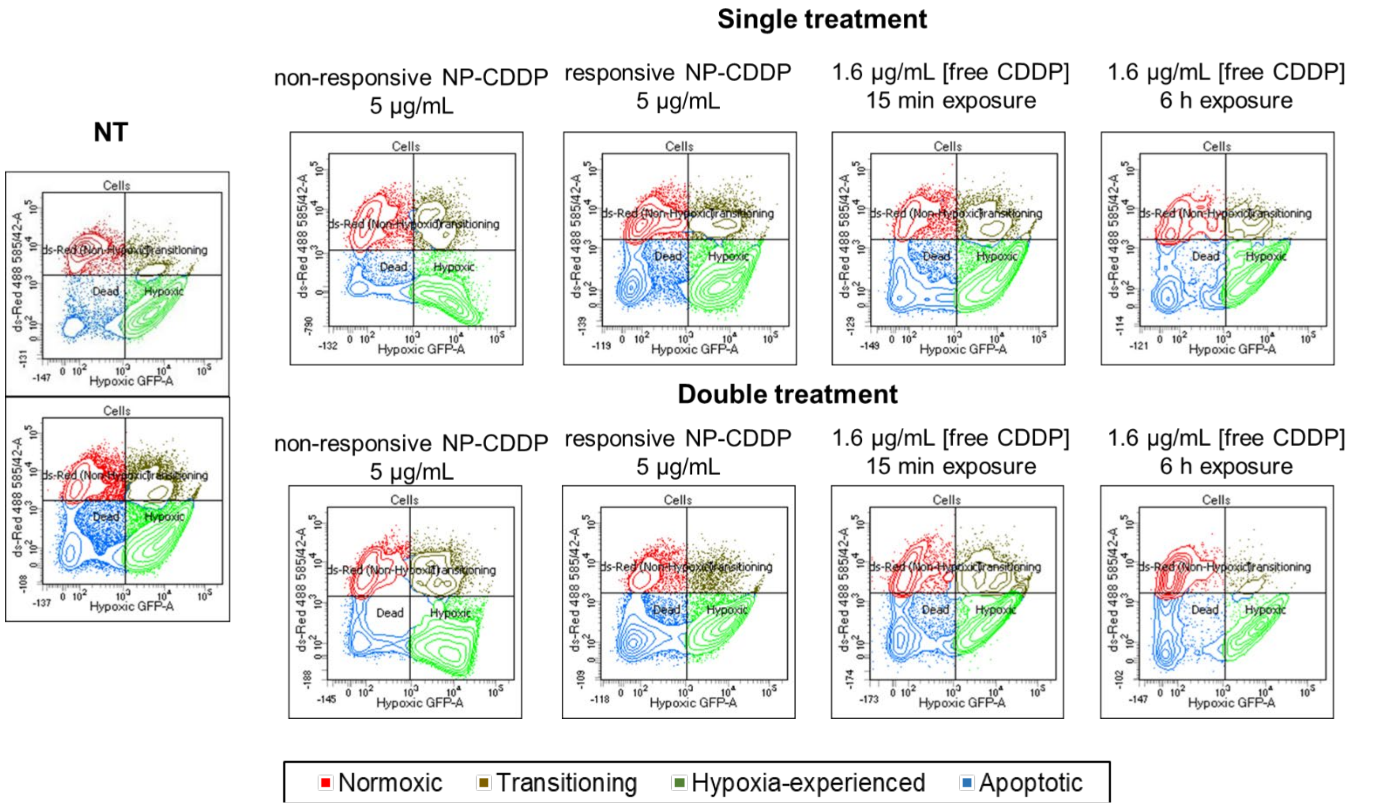

Figure S5. Characterization of cell populations by flow cytometry after treatment. Sample of Flow Cytometry measurements, to quantify the cell population profiles, following different treatments as indicated. Cells that have not experienced hypoxia are shown in red, cells that are transitioning or partially exposed to hypoxic conditions are shown in brown, cells that have experienced hypoxic conditions are indicated by green, and cells that have undergone apoptosis are indicated by blue.

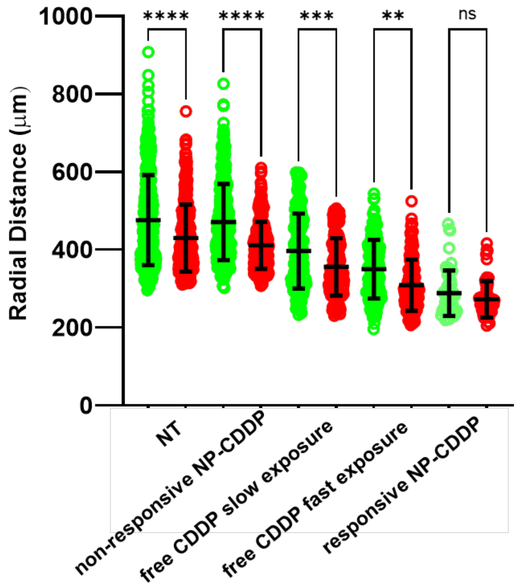

Figure S6. Effect of treatment(s) on the migration of cells from spheroids. Effect of different CDDP treatments on the migration of cells from spheroids. Radial locations of cells that migrated, from the spheroid into the collagen gel, relative to the spheroid center (migration distance), at the same time point of 40 hours as shown in Figures 7a and 7b. Cells in normoxic conditions are shown in red, and cells that have experienced hypoxic conditions in green. Data collected from n=3 spheroids per treatment. Horizonal lines indicate the mean and standard deviation averaged over the entire cell counts. p-values: **<0.01, ***<0.001, ****<0.0001. NT = not treated spheroids.
